# Circadian Rhythm of Wrist Temperature among Shift Workers in South Korea: A Prospective Observational Study

**DOI:** 10.3390/ijerph14101109

**Published:** 2017-09-24

**Authors:** Tae-Won Jang, Hyunjoo Kim, Suk-Hoon Kang, Sang-Hyo Choo, In-Seok Lee, Kyung-Hwa Choi

**Affiliations:** 1 Department of Occupational and Environmental Medicine, Hanyang University, Seoul 04763, Korea; om1024@hanmail.net; 2 Department of Occupational and Environmental Medicine, Ewha Womans University Mokdong Hospital, Seoul 07985, Korea; 3 Center for Sleep Medicine, Veterans Health Service Medical Center, Seoul 07985, Korea; sukhoon.kang@yonsei.ac.kr; 4 Department of Occupational and Environmental Medicine, Sojung Healthcare, Seoul 05368, Korea; elfhunt2000@daum.net; 5 Department of Civil, Safety and Environmental Engineering, Hankyong National University, Anseong 17579, Korea; lis@hknu.ac.kr; 6 Taean Environmental Health Center, Taean 32148, Korea; rosach72@hanmail.net

**Keywords:** circadian rhythm, shift work, body temperature, wrist temperature, Cosinor analysis

## Abstract

*Background*: Human body temperature varies with circadian rhythm. To determine the effect of shift work on the circadian rhythm of the distal-skin temperature, wrist temperatures were measured. *Methods*: Wrist-skin temperatures were measured by an iButton^®^ Temperature Logger. It was measured every 3 min for two and eight consecutive working days in the day and shift workers, respectively. Mesor, amplitude, and acrophase were measured by Cosinor analysis. *Results*: The shift-worker amplitude dropped significantly as the night shift progressed (0.92 to 0.85 °C), dropped further during rest (0.69 °C), and rose during the morning-shift days (0.82 °C). Day workers still had higher amplitudes (0.93 °C) than the morning-shift workers. The acrophase was delayed during the four night-shift days, then advanced during rest days and the morning-shift days. Nevertheless, the morning-shift worker acrophase was still significantly delayed compared to the day workers (08:03 a.m. vs. 04:11 a.m.). *Conclusions*: The further reduction of wrist-temperature amplitude during rest after the night shift may be due to the signal circadian rhythm disruption. Reduced amplitudes have been reported to be associated with intolerance to shift work. The findings of our study may help to design the most desirable schedule for shift workers.

## 1. Introduction

Many physiological processes such as the sleep-wake cycle, locomotor activity, body temperature, hormone secretion, and metabolism change regularly during each day/night cycle. These physiological changes are called circadian rhythms [1]. The period of these circadian rhythms is approximately 24 h and is controlled by the circadian clock or internal body clock [2]. The circadian clock is shaped by zeitgebers, which are environmental cues such as light that indicate the external time.

In usual, circadian rhythm is programmed for being active during the day and sleep during the night. Shift workers should change the timing of wake and sleep times, and this schedule does not match their internal body clock [3]. This mismatch between the timing of the internal body clock and environmental timing is known as external desynchronization. When this mismatch occurs, their circadian rhythm starts to follow the new sleep and wake cycle to synchronize internal clock to environmental time. During this process, the circadian rhythm system is not working efficiently, and this is known as internal desynchronization or circadian disruption [4].

The subject of this study is one of these circadian rhythms in humans, namely, body temperature. The core body temperature is usually lowest at the end of the sleep phase and highest in the late afternoon [5]. Thus, over a 24 h period, the oral temperature ranges from 33.2 to 38.2 °C, the rectal temperature ranges from 34.4 to 37.8 °C, and the axillary temperature ranges from 34.7 to 37.5 °C [6]. The distal temperature of the body also changes during the 24 h circadian cycle: Sarabia et al. [7] reported that the wrist temperature changed by 0.433 °C in the working week and 0.884 °C in the holidays.

Both the core body temperature and the distal temperature are determined by the amount of heat production by the core and the amount of heat radiated from the distal part of the body. Significantly, both heat production and heat loss change during the 24 h circadian cycle, thus leading to differences in core and distal body temperatures [8]. In relation to changes in heat loss, heat radiation through the skin rises at dawn and drops in the late afternoon. As a result, the distal-skin temperature peaks at dawn and reaches its nadir in the late afternoon. This also affects the core body temperature, which is lowest at dawn and highest in the late afternoon [9].

The sleep-wake cycle relates closely to these circadian body temperature changes. Individuals usually fall asleep when the core body temperature decreases and wake when the core body temperature rises [10,11]. This relationship between sleep and body temperature led us to ask whether shift workers, whose sleep-wake cycle is determined by when they work, differ from day workers in terms of the circadian rhythm of their body temperature. Previous research reported that body temperature amplitude had decreased during night shift work, and low amplitude had been related with poor tolerance of shift work. However, there was no study on the change of body temperature during rest day and morning shift after night shift [12,13]. We hypothesized that the circadian rhythm of body temperature would change and the amplitude would be different according to the timing of work shift in shift workers. To identify this, we measured the distal-skin temperature of day and shift workers and determined the effect of changing shifts on the circadian rhythm of distal temperature.

## 2. Materials and Methods

### 2.1. Study Design and Ethics

All participating subjects provided written informed consent after the objectives and methods of the study were explained to them. The study was approved by the Institutional Review Board of the Veterans Health Service Medical Center (approval ID: 2016-07-016-001). The study was conducted according to the tenets of the Helsinki Statement and its revisions.

### 2.2. Subjects

All subjects were adult employees of a single manufacturing company who routinely worked either during the day or in shifts and who volunteered to participate in the study. The study started in July 2016 and was completed 3 months later (September 2016). The company manufactures semiconductors and is located in Icheon, in the Gyeonggi province of South Korea. Most shift workers in this company were manual workers. The subjects were recruited by study recruitment announcements that were posted on the bulletin board of the company. Subjects were excluded if they were pregnant or had medical problems such as infectious diseases. In addition, subjects who were found not to have worn the skin temperature-measuring device properly were excluded from analysis. In total, 68 day workers and 53 shift workers participated in the study. Table 1 shows the demographic characteristics of the two groups and their employment duration.

### 2.3. Questionnaire and Wrist-Skin Temperature Measurements

Before commencing skin temperature measurements, all subjects completed a self-report questionnaire that asked about age, gender, height, weight, education, marital status, and duration of employment at the manufacturing company. The subjects then received an iButton^®^ Temperature Logger (iButton^®^ DS1922L, Maxim Integrated Products, Inc., San Jose, CA, USA), which is a computer chip enclosed in a 16-mm-thick stainless steel can. The device was attached to the palmar side of the wrist of the subject’s non-dominant hand using 3MTM micropore surgical tape. We requested that the subjects not take off iButton^®^ except when exposed to water, such as showering. If they needed to take off iButton^®^ for showering, they were asked to wear it in the same place after the shower. The iButton^®^ DS1922L can measure temperatures ranging from −40 to +85 °C, and the resolution is 0.0625 °C.

**Figure 1:**
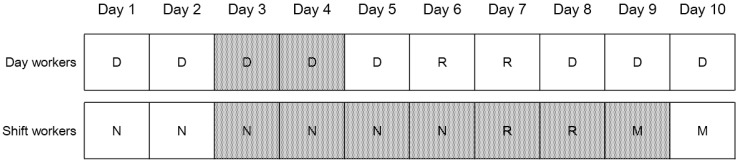
The schedule for wrist-skin temperature measurement. Grey grid means the measurement day, D, R, N, and M means day work, rest day, night shift work, and morning shift work, respectively.

The day workers at the company worked from 08:00 a.m. to 05:00 p.m. The shift workers worked the morning shift (06:00 a.m.–02:00 p.m.), followed by the afternoon shift (02:00–10:00 p.m.), and then the night shift (10:00 p.m.–06:00 a.m.). Each 8 h shift was performed 6 days in a row and included a break of 40 min for mealtimes. Every 6 days shift period was followed by a 2 days rest interval. The wrist temperature of all day workers was measured every 3 min during two consecutive working days. The wrist temperature of all shift workers was measured every 3 min for eight consecutive days in which the employees worked four night shifts (from 3rd to 6th night shift), had two rest days, and then worked two morning shifts (Figure 1). We recommended that they refrain from alcohol drinking and intense exercise during the measurement period.

After the measurements had been completed, the data stored in the iButton^®^ were downloaded to a computer via a USB adapter (DS9490B) using the software OneWire Viewer version 3.2 (Maxim Integrated Products, Inc., San Jose, CA, USA). Since the raw temperature data contained some errors such as those generated by temporarily removing the iButton^®^, we removed all extreme temperature measurement data before analysis.

### 2.4. Temperature Analyses

Cosinor analysis [14] was used to analyze the circadian rhythm of wrist temperature in each subject. For this, the software Cosinor version 3.1 (Circadian rhythm laboratory, Boise, ID, USA), which can be downloaded gratis from the website of Refinetti [15], was used. The following circadian variables were extracted from the temperature data: the mesor (the circadian rhythm-adjusted mean temperature, which is based on the parameters of a cosine function), the amplitude (the difference between the temperature peak and the temperature mesor of a cosine function), and the acrophase (the time point at which the circadian temperature peak occurred) [16]. Cosinor version 3.1 included an F test to evaluate whether the amplitude of a cosine wave fitted to the data is significantly greater than zero [17].

### 2.5. Statistical Analyses

The day and shift workers were compared in terms of demographics and duration of employment by using a Student’s *t*-test and a chi-square test. The average ± standard deviation of the amplitude, mesor, and acrophase of the day workers during their two work days were calculated. These averages were also calculated for the shift workers for Night-Shift Days 3–4, Night-Shift Days 5–6, the two rest days, and Morning-Shift Days 1–2. The average acrophase was expressed as 12 h clock time point. The day workers were compared to the shift workers in terms of mesor, amplitude, and acrophase by using Student’s *t*-test. For this comparison, the morning-shift data of the shift workers was used. In addition, the shift-worker data were analyzed by using repeated-measures analysis of variance (ANOVA) to determine whether the circadian rhythm variables changed when the workers moved from the night shift to rest to the morning shift. All statistical analyses were conducted by using SAS windows version 9.4 (SAS Institute Inc., Cary, NC, USA). The statistical significance level was set at *p* < 0.05.

## 3. Results

Table 2 shows the results of the Cosinor analysis of the wrist-skin temperatures that were measured over 2 days in the day workers and the two morning-shift days of the shift workers. The shift workers had significantly lower temperature amplitudes than the day workers and their acrophase was significantly delayed by about 4 h (*p* < 0.05). The two groups did not differ in terms of mesor. The males and females in the groups had similar patterns to the whole cohort.

Table 3 shows the Cosinor analysis results of the wrist-skin temperatures of the shift workers over the eight consecutive days of measurement. The mesor temperature of the shift workers did not change during the study period. In terms of temperature amplitude, it was 0.92 °C on Night-Shift Days 3–4 and dropped to 0.85 °C on Night-Shift Days 5–6. During rest, the amplitude dropped further to 0.69 °C. However, during Morning-Shift Days 1–2, the amplitude rose to 0.82 °C. These changes were significant on repeated-measures ANOVA (*p* < 0.05).

The acrophase was 11:59 a.m. in Night-Shift Days 4–5 and 23 min later in the fifth and sixth night-shift days (12:22 p.m.). It then advanced by 2 h during rest (10:27 a.m.), and then advanced further by almost 2.5 h during the morning-shift days (08:03 a.m.).

## 4. Discussion

In the present study, the distal-skin temperatures of day and shift workers in a manufacturing company were measured. The principal findings are as follows. First, the amplitude of the day workers was 0.93 °C, which was higher than all of the amplitudes measured in the shift workers. There was also a steady decrease in shift-worker amplitude as the night shift progressed (from 0.92 to 0.82 °C), and the lowest amplitude was observed during the rest days (0.69 °C). During the morning shift, however, the amplitude rose to 0.82 °C. Second, the acrophase of the day workers was 04:11 a.m. By comparison, night shift was associated with a delay in acrophase. However, during rest and then the morning shifts, the acrophase advanced rapidly.

Several studies have shown that shift work changes the amplitude of body temperature. One was by Härmä et al. [18], who reported that night-shift work decreases the oral temperature amplitude. Notably, Reinberg et al. [19] reported that small amplitudes in oral temperature are associated with intolerance to shift work, whereas large amplitudes are associated with tolerance. These findings were confirmed by Andlauer et al. [12] and Knauth et al. [13]. Moreover, Nesthus et al. [20] showed that, compared to clockwise-rotating shift work (two morning shifts, two evening shifts, and one night shift followed by two rest days), counterclockwise-rotating shift work (two evening shifts, two morning shifts, and one night shift followed by two rest days) associated with greater attenuation of body temperature amplitude. Ferreira et al. [21] reported that amplitude reduction had been observed in nursing students during nocturnal work. In the present study, the wrist-temperature amplitude among shift workers completing the first 2 days of a 6 days of morning shifts was lower than the amplitude of day workers. This is consistent with the previous studies. We also found that males and females were similar in terms of the effect of shift work on wrist-temperature amplitude.

We observed that, while the amplitude during the third and fourth days of the 6 day night-shift period was only slightly lower than the amplitude of the day workers (0.92 °C vs. 0.93 °C), it gradually decreased during the subsequent night-shift days and the rest period. Notably, the amplitude was lowest on the rest days. However, during the subsequent morning-shift days, the amplitude rose again. When Knauth and Rutenfranz [22] measured rectal temperature during 21 consecutive night shifts in six subjects aged 19–28 years, they found that amplitude reduction had been observed in the first week of night shifts, and amplitude reduction had vanished from the 6th night shift. The amplitude had not been significantly different from day work in the subsequent 14 days. These findings suggest that the synchronization of the circadian rhythm starts with the night-shift and that, if the night-shift period is long enough (i.e., at least 7 days), the amplitude will normalize to the levels seen in day workers. However, in the present study, amplitude reduction was observed in 4 night shifts and *w* rest days. The synchronization between internal clock and environmental timing (night shift) would progress continuously after the 5th night shift in the study of Knauth and Rutenfranz [22], and their circadian rhythm would be stabilized more and more. However, in the present study, the synchronization would stop and synchronization toward the opposite direction would start after the 6th night shift, so their circadian rhythm would be disrupted again. This may account for the difference between the study of Knauth and Rutenfranz [22]. Notably, the rest period was also associated with a marked advancement (by 2 h) of the acrophase. This suggests that, during the rest period, there was rapid disruption of the circadian rhythm that was being established during the night-shift period. This may explain why the lowest amplitude of wrist temperature was observed on the rest days.

The circadian rhythm is changed by the exposure to bright light, which can either advance the acrophase or delay it [23]. Consequently, shift work changes the circadian rhythm. Thus, workers with clockwise-rotating shifts (i.e., morning shift followed by evening shift followed by night shift followed by rest) will exhibit successive delays in their acrophase as the work period progresses. By contrast, when shift workers rest after the night-shift period, their acrophase will advance, thereby normalizing to the acrophase seen in day workers. These observations are supported by the study by Härmä et al. [18], who found that nurses who worked a morning shift followed by two consecutive night shifts (i.e., a counterclockwise shift schedule) exhibited a shift forward in the oral temperature acrophase. Moreover, in the study of Knauth et al. [24], shift workers who worked 7 days of night shift after a rest exhibited a delay in acrophase from 4:30 p.m. in the first night shift to 7:30 p.m. in the seventh night shift. In the present study of workers with a clockwise shift-work schedule separated by 2 day rest intervals, the acrophase was delayed to 11:59 a.m. and 12:22 p.m. during the night shift, advanced to 10:27 a.m. during the rest period, and then further advanced to 8:03 a.m. during the morning shift.

In the present study, the day workers started working at 08:00 a.m., whereas the morning-shift workers started working at 06:00 a.m. Since the morning-shift workers would at least theoretically wake up earlier than the day workers, their circadian phase should be advanced relative to that of the day workers. However, we found that the circadian phase of the morning-shift workers, as indicated by the wrist-skin temperature, was delayed by 4 h, rather than being advanced. This reflects the fact that the shift workers had had six night shifts followed by a 2 day rest before they started the morning shift. Synchronization of the circadian rhythm with the 6 day night-shift period had caused their circadian phase to be delayed. Although the circadian phase was advanced during the two rest days after the night shifts, it is likely that the synchronization of the circadian rhythm was not completed during the two rest days. As a result, the circadian rhythm of the shift workers was still delayed in the first and second morning-shift days. Such delays in the circadian rhythm may explain why many shift workers feel fatigue during the morning shift and appear to be more intolerant to morning-shift work [25,26].

The mesor is the circadian rhythm-adjusted mean that is based on the parameters of a cosine function. The body temperature mesor varies depending on where body temperature is measured. Thus, when the mesor of the core body temperature is 37.19 °C, the mesor of the rectal and axillary temperature is 36.01 °C [27]. Moreover, the wrist-temperature mesor is usually lower than the rectal and axillary temperature mesor. Indeed, Sarabia et al. [7] found that the wrist-temperature mesor was 34.034 °C during the holiday period and 33.967 °C during the working week. Bracci et al. [28] observed that, compared to day nurses, shift-working nurses had a lower distal-skin temperature mesor, an unchanged maximum temperature, and a higher minimum temperature; as a result, the shift workers had a smaller temperature amplitude than the day nurses. In the present study, the wrist-temperature mesor on the rest days of the shift workers tended to be higher than the mesor during the night shift, although this difference did not achieve statistical significance. This observation may also be due to the reduction in amplitude, which is thought to be associated with disruption of the circadian rhythm.

The present study has several limitations. First, wrist-skin temperature may be affected by several factors such as physical activity, environmental temperature, and sleep [29,30]. Shift workers engaged in a variety of jobs, but the physical demand was similar to each other. To control physical activity outside of their work, we asked study subjects to refrain from intense exercise during the measurement period. Different sleep-wake patterns is an important feature for shift work, and circadian disruption in shift workers is due to different sleep-wake patterns, as well as shift work schedule. Therefore, it would not be desirable to control sleep-wake patterns of shift workers. Therefore, although we did not consider the factors associated with wrist-skin temperature such as physical activity and sleep-wake patterns, the effect of them may not be significant. Second, the menstrual cycle of women can affect their body temperature: in the luteal phase, the body temperature rises and the body temperature amplitude drops [31]. We did not consider the menstrual cycle of the female subjects when analyzing the data in the present study. Third, we only studied one type of shift-work schedule. It is not clear whether the findings of the present study can be extrapolated to the many other types of shift-work schedules that exist.

Despite these limitations, the present study has several strengths. First, we measured the wrist temperature every 3 min over eight consecutive days that included four night shifts, two rest days, and two morning shifts. This allowed us to closely observe the variation in the wrist temperature during several parts of the shift-work schedule. The inclusion of measurements during the rest days led to a significant finding, namely, that the reduction in the body temperature amplitude may be associated with disruption of the circadian rhythm.

## 5. Conclusions

In summary, we measured the wrist temperature in day and shift workers and found that the night-shift workers had lower wrist-temperature amplitudes than the day workers. We also found that the amplitude was lowest on the subsequent rest days. Thus, reduction in the wrist-temperature amplitude, which has been reported to be associated with intolerance to shift work [17,18,19], may be associated with disruption of the circadian rhythm. In addition, the reduction may worsen during rapid disruption of the circadian rhythm. The findings of our study may help to design the most desirable schedule for shift workers.

## Figures and Tables

**Table 1:** Demographic characteristics of the day and shift workers.

Variables	Day Workers (*n* = 68)	Shift Workers (*n* = 53)
Age, years		
<30	31 (45.6)	28 (52.8)
30–39	31 (45.6)	21 (39.6)
≥40	6 (8.8)	4 (7.6)
Gender		
Male	36 (52.9)	16 (30.2)
Female	32 (47.1)	37 (69.8)
Duration of employment, years		
<10	48 (70.6)	35 (66.0)
10–19	16 (23.5)	14 (26.4)
≥20	4 (5.9)	4 (7.6)

The data are expressed as number and frequency (%).

**Table 2:** Cosinor analysis of the wrist-skin temperature of the day and shift workers.

Group	Mesor, °C	Amplitude, °C	Acrophase
Total			
Day workers	33.8 ± 0.4	0.93 ± 0.40 ^2^	04:11 ± 02:59 a.m. ^2^
Shift workers ^1^	33.9 ± 0.5	0.82 ± 0.36 ^2^	08:03 ± 05.05 a.m. ^2^
Male			
Day workers	33.8 ± 0.4	0.94 ± 0.41 ^2^	04:16 ± 03:05 a.m. ^2^
Shift workers ^1^	33.8 ± 0.5	0.83 ± 0.26 ^2^	07:30 ± 05:09 a.m. ^2^
Female			
Day workers	33.8 ± 0.4	0.92 ± 0.40 ^2^	04:06 ± 02:55 a.m. ^2^
Shift workers ^1^	34.0 ± 0.5	0.81 ± 0.42 ^2^	08:24 ± 05:07 a.m. ^2^

The data are shown as mean ± standard deviation or 12 h clock time. ^1^ The temperature data were from the shift workers during the first 2 days of the morning shift; ^2^ Significantly different between day and shift workers (*p* < 0.05), as determined by Student’s *t*-test.

**Table 3:** Cosinor analysis of the wrist-skin temperature among shift workers.

Time of Measurement	Mesor (°C)	Amplitude (°C)	Acrophase
3rd and 4th night shift	33.9 ± 0.4	0.92 ± 0.36	11:59 ± 05:35 a.m.
5th and 6th night shift	33.8 ± 1.5	0.85 ± 0.33 ^1^	12:22 ± 04:33 p.m. ^1^
2 rest days	34.1 ± 0.5	0.69 ± 0.34 ^1^	10:27 ± 05:57 a.m. ^1^
1st and 2nd morning shift	33.9 ± 0.5	0.82 ± 0.36 ^1^	08:03 ± 05:05 a.m. ^1^

The data are shown as mean ± standard deviation or 12 h clock time. ^1^ Significantly different compared to the 3rd and 4th night-shift data, as shown by repeated-measures ANOVA.
